# Tertiary amine mediated aerobic oxidation of sulfides into sulfoxides by visible-light photoredox catalysis on TiO_2_ †
†Electronic supplementary information (ESI) available. See DOI: 10.1039/c5sc01813g



**DOI:** 10.1039/c5sc01813g

**Published:** 2015-06-09

**Authors:** Xianjun Lang, Wei Hao, Wan Ru Leow, Shuzhou Li, Jincai Zhao, Xiaodong Chen

**Affiliations:** a School of Materials Science and Engineering , Nanyang Technological University , 50 Nanyang Avenue , Singapore 639798 , Singapore . Email: lisz@ntu.edu.sg ; Email: chenxd@ntu.edu.sg; b Key Laboratory of Photochemistry , Beijing National Laboratory for Molecular Sciences , Institute of Chemistry , Chinese Academy of Sciences , Beijing 100190 , China

## Abstract

The selective oxidation of sulfides into sulfoxides receives much attention due to industrial and biological applications. However, the realization of this reaction with molecular oxygen at room temperature, which is of importance towards green and sustainable chemistry, remains challenging. Herein, we develop a strategy to achieve the aerobic oxidation of sulfides into sulfoxides by exploring the synergy between a tertiary amine and titanium dioxide *via* visible-light photoredox catalysis. Specifically, titanium dioxide can interact with triethylamine (TEA) to form a visible-light harvesting surface complex, preluding the ensuing selective redox reaction. Moreover, TEA, whose stability was demonstrated by a turnover number of 32, plays a critical role as a redox mediator by shuttling electrons during the oxidation of sulfide. This work suggests that the addition of a redox mediator is highly functional in establishing visible-light-induced reactions *via* heterogeneous photoredox catalysis.

## Abstract 2

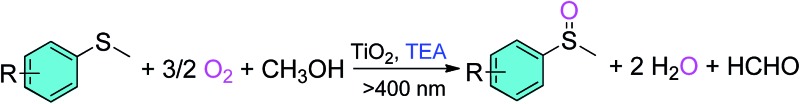
The selective aerobic oxidation of sulfides into sulfoxides on TiO_2_ under visible-light irradiation was accomplished through synergistic catalysis with triethylamine.

## Introduction

The selective oxidation of sulfides into sulfoxides, an essential chemical transformation, plays a pivotal role in accessing a series of industrially and biologically important organic compounds.^1–5^ In line with the principles of green chemistry for selective oxidation, both O_2_ (ref. 1–3) and H_2_O_2_ (ref. 4 and 5) can be used as the oxidant for such a specific reaction with the only side product being water. Using O_2_ as the oxidant proves extremely challenging due to the difficulty of O_2_ activation and its subsequent highly reactive nature once activated.^6–10^ These issues can be resolved by visible-light photoredox catalysis, which can ensure the activation of O_2_ under mild conditions and tame the reactive oxidative species to yield sulfoxide products.^11–13^ To this end, the reaction has been successfully carried out in homogeneous photocatalytic systems using organic dyes such as Rose Bengal and the [Ru(bpy)_3_]^2+^ complex,^14–17^ as well as heterogeneous photocatalytic systems using metal–organic frameworks, CdS and metal-free organic polymers.^18–24^ With so many precedents, it is odd that the quintessential photocatalyst TiO_2_ (ref. 25–32) was not as successful as we expected in achieving the selective oxidation of sulfides.

The low success rate of TiO_2_ could be attributed to the fact that typical sulfides like thioethers contain aliphatic side chains, which can be fragmented by the TiO_2_-induced free radical process, therefore leading to sulfoxides in low selectivity. In some reported systems, protic solvents have been employed to prevent this undesired side reaction. However, this could not be extended to a TiO_2_-based system because the high oxidation potential (2.7 V *vs.* NHE for anatase) of a UV-induced hole results in the oxidation of protic solvents rather than the sulfides. In addition, the large band gap (3.2 eV for anatase) indicates that TiO_2_ could not absorb visible-light directly.

To surmount the innate lack of visible-light absorption and preserve the intrinsic high activity of TiO_2_, the formation of a surface complex with organic substrates can be used to enable visible-light activity and establish selective photoredox catalytic processes.^33–42^ In detail, the Lewis acid sites of TiO_2_ interact with the lone pairs of heteroatoms such as N, O and S within a substrate to form a surface complex and extend the absorption edge of TiO_2_. Thus no UV-induced holes are involved in the redox process. O_2_ can be activated in a tightly controlled manner only in the presence of the target substrate after the occurrence of this interaction. Furthermore, the conduction band of TiO_2_ also facilitates electron transfer to O_2_. This approach not only enables visible-light, which is predominant in the solar spectrum, to be efficiently utilized, but also avoids UV-initiated side reactions, resulting in high selectivity for the desired product. Recently, we have successfully demonstrated this strategy through the synergistic photocatalytic oxidation of sulfides and amines by O_2_ on TiO_2_ under visible-light irradiation.^43^ In that reaction system, the oxygenation of sulfides by O_2_ was aided by the presence of primary benzylic amines.

In the aforementioned system, the primary benzylic amines also reacted, which could potentially complicate the separation of products and perplex the understanding of the reaction mechanism. In addition, the presence of benzylic amines results in further reactions, which bewilders the mechanistic understanding; and these relatively large molecules make computational studies much more time-consuming. In contrast, a tertiary amine will not undergo further condensation reactions, so we hypothesized that a non-benzylic aliphatic tertiary amine might remain stable in the catalytic cycle to further mediate the oxidation of sulfides. Thus, only a catalytic amount of tertiary amine would be needed to execute the oxidation process. Using a small molecular tertiary amine is also recommended in practice to facilitate the easy separation of catalyst and product. Not only that, if the tertiary amine remains intact at the end of the reaction, the in-depth understanding of the photochemical mechanism could be mapped out more precisely since no assumptions are needed to figure out a computationally plausible mechanism at as high a level of theory as is practically possible. As a result, it is of interest to study photoredox synergistic catalysis by TiO_2_ and a tertiary amine in the selective oxidation of sulfides using O_2_.

**Scheme 1:**
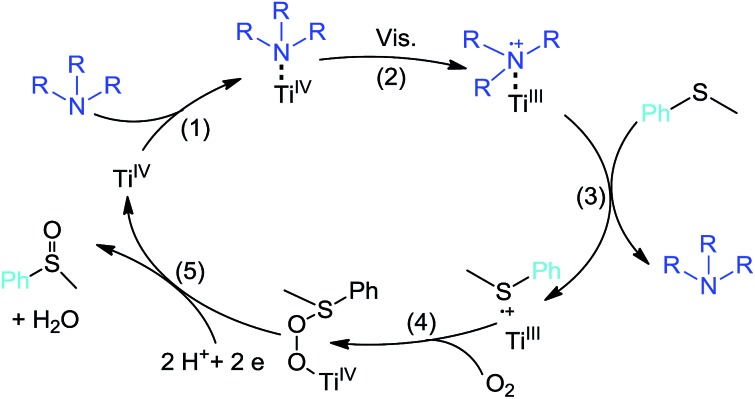
Proposed mechanism for the aerobic oxidation of thioanisole on TiO_2_ with tertiary amine as the redox mediator under visible-light irradiation.

Trimethylamine (TMA) and triethylamine (TEA) are small molecular tertiary amines which are widely used as reductants in visible-light-induced reduction reactions.^44^ They have also been used as model pollutants to investigate the TiO_2_ photodegradation process.^45,46^ They are not considered stable under oxidation conditions, and thus are rarely used as co-catalysts in aerobic oxidations. The problem of instability of TMA or TEA can be circumvented by the reaction environment, such as the solvent used. The selection of nitrogen-containing molecules to enable visible-light photoredox reactions is quite reasonable considering that nitrogen-doping is the most successful method for sensitizing TiO_2_ to visible-light photocatalysis.^47^ We should clarify that “surface adsorption” and “bulk doping” are totally different tactics for achieving visible-light activity. However, underpinning conclusions can be drawn from each tactic and some researchers even propose “surface adsorption” as “*in situ* doping”.^48^ This is reinforced by the suggestion that the process of nitrogen-doping might have originated from the formation of a surface complex on the surface of TiO_2_.^49^ The hypothesized photoredox synergistic catalysis is simply described in Scheme 1: (1) the tertiary amine interacts with the vacant Ti sites on the surface of TiO_2_ to form a visible-light absorbing surface complex; (2) single electron transfer from the amine to the conduction band of TiO_2_ occurs under visible-light irradiation, leaving the hole localized on the amine to form a cationic free radical; (3) the amine cationic free radical is captured by sulfide to produce a sulfide cationic free radical, restoring the amine; (4) oxygen-atom transfer to sulfide occurs, replenishing the Ti^IV^ state; and (5) further electron and proton transfers aid the formation of the product, which leaves the Ti site and thus completes the entire photoredox catalytic cycle.

## Results and discussion

To confirm the aforementioned mechanism, the interaction between the amine and TiO_2_ (evidenced by XPS, Fig. S1†), which is crucial for the observed visible-light activity in Scheme 1, should be identified. We employed first-principles calculations based on density functional theory to investigate the adsorption of the amine onto the Degussa P25 TiO_2_ surface, which consisted of 25% rutile and 75% anatase morphologies. For computational expediency, simulations were based on a model of the smaller TMA molecule using both the (110) surface of rutile and the (101) surface of anatase, which constitute the most stable surfaces for rutile and anatase TiO_2_.^50,51^

**Fig. 1:**
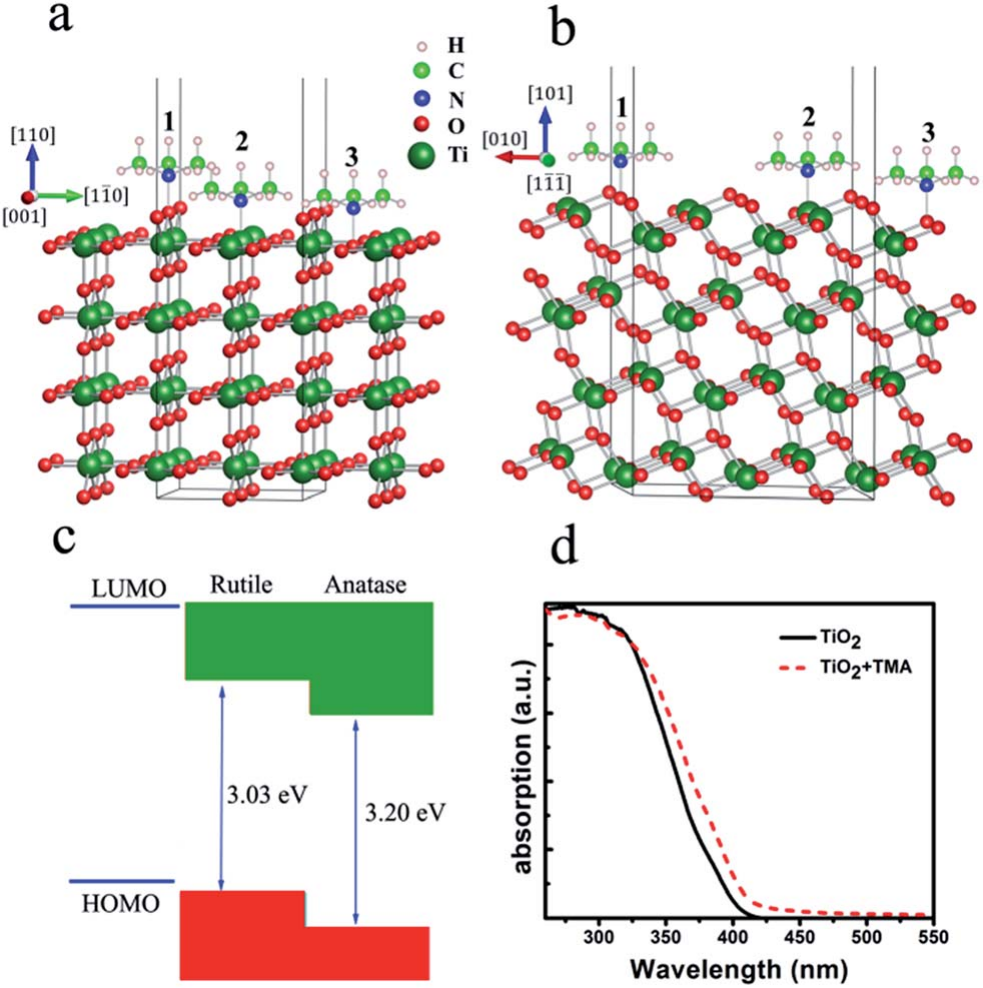
The formation of a surface complex of TiO_2_ and its implications: a and b, the possible adsorption sites of TMA onto the rutile (110) and anatase (101) surfaces of TiO_2_; c, schematic (not to scale) of the band alignment of TMA onto the surface of Degussa P25 TiO_2_; d, UV-visible absorbance spectroscopy of Degussa P25 TiO_2_ and the complex between TMA and TiO_2_.

Following the setup of the surface models (see ESI† for details), the adsorption of TMA can be calculated in a straightforward way. The adsorption energy *E* ^ad^ was calculated according to the following equation:*E* ^ad^ = *E* ^TMA/slab^ – *E* ^slab^ – *E* ^TMA^where *E* ^TMA/slab^, *E* ^slab^ and *E* ^TMA^ are the total energies of the slab with TMA, the slab alone, and TMA, respectively. A negative adsorption energy indicates that TMA can be absorbed onto the slab surface. We have examined three adsorption sites for TMA onto the rutile (110) and anatase (101) surfaces; the O-2c site (**1**), Ti-5c site (**2**) and O-3c site (**3**) (Fig. 1a and b). The calculated adsorption energies are listed in Table 1. From the results in Table 1, we can see that the Ti-5c sites for both surfaces (Fig. S3†) are more favorable for the adsorption of the TMA molecule, which is in excellent agreement with step (1) proposed in Scheme 1. The adsorption energy of TMA onto the Ti-5c site (**2**) of the rutile (110) surface is –0.43 eV, which is smaller than that of the anatase (101) surface (–0.28 eV), indicating that the adsorption of TMA onto rutile (110) is preferable to that onto the anatase (101) surface. The distances from the N atom to the nearest Ti atom are 2.79 Å for the rutile (110) surface and 2.44 Å for the anatase (101) surface, indicating that TMA is physically adsorbed onto these two surfaces. The binding force between TMA and TiO_2_ is a Lewis base and acid interaction. Thus, the visible-light absorption of the surface complex is in a quite narrow range; and the desorption of TMA from the TiO_2_ surface occurs easily. This result agrees well with step (3) proposed in Scheme 1 in which the N-centered cationic radical could be replaced by the S-centered cationic radical.

**Table 1:** The calculated adsorption energies *E* ^ad^ (eV) of TMA onto the anatase (101) and rutile (110) surfaces

Surface	O-2c (1)	Ti-5c (2)	O-3c (3)
Rutile (110)	–0.04	–0.43	–0.09
Anatase (101)	2.20	–0.28	–0.04

Based on the above calculation results, TMA should be localized on the surface of rutile TiO_2_. Using a recent report on the band alignment between rutile and anatase in Degussa P25 TiO_2_,^52^ the electron transfer between TMA and Degussa P25 TiO_2_ can be described as in Fig. 1c. In Degussa P25 rutile the valence band *E*_vb_ = 2.31 V (*vs.* NHE). With the adsorption of TMA, the band gap of rutile TiO_2_ does not change, but calculations reveal an increase in electron density (Fig. S4†). However, the UV-visible absorption showed a red-shift of about 10 nm (Fig. 1d), suggesting a narrowing of the band gap by 0.08 V, which may have been too small to be reflected by the calculations. This suggests that the *E*_HOMO_ of the adsorbed TMA surface-complex is about 2.23 V (*vs.* NHE). The single electron oxidation potential of thioanisole in CH_3_OH is 1.75 V (*vs.* NHE),^53^ suggesting that the oxidation potentials are well matched to allow a smooth electron transfer process. Specifically, under visible-light irradiation, the electron in the HOMO of the adsorbed TMA will be excited, leaving a hole for the oxidation of thioanisole. Meanwhile, the excited electron will be injected into the conduction band of rutile, it will then be further transferred to the conduction band of anatase, and ultimately to the electron acceptor, which can be either O_2_ or the surface-bound peroxide in step (4) of Scheme 1.

To verify our hypothesis, we conducted the aerobic sulfide oxidation with a variety of small molecular amines with low boiling points, which would be easy to separate from the sulfoxide product. The experimental results are presented in Table 2. The conversion of thioanisole in CH_3_OH was very low under visible-light irradiation (entry 1, Table 2). Interestingly, with the introduction of a catalytic amount (0.1 equiv.) of amine into the reaction system, the conversion of thioanisole was significantly boosted (entries 2–6, Table 2). Like in our previous report, primary amines can initiate the oxidation (entries 2–3, Table 2), and a decrease in the amount of amine leads to a decrease in the conversion of thioanisole (entry 7, Table 2). As expected, the tertiary amines TMA and TEA remained stable during the oxidation process, ensuring higher conversions of thioanisole (entries 5 and 6, Table 2). However, the installment of a functional group in the side chain of TEA, such as in triethanolamine (TEOA), would make TEOA unstable in the reaction conditions. Fragmentation of TEOA dominates rather than it performing its duty as a redox mediator, leading to a sharp decrease in the conversion of thioanisole (entry 8, Table 2). Thus, besides enhancing the visible-light activity, TEA plays a more significant role as a metal-free redox mediator by shuttling electrons during the oxidation of sulfide, which is analogous to how transition metal ions such as Ni^2+^ or Ru^3+^ act as redox shuttles in prompting the photocatalytic reduction reaction.^54,55^

**Table 2:** The influence of amine on the selective aerobic oxidation of thioanisole under visible-light irradiation^*a*^

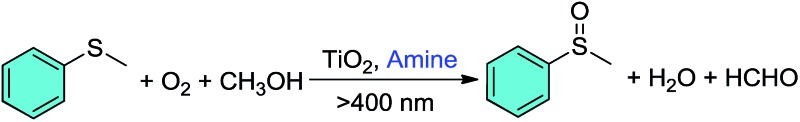			
Entry	Amine	Conv.^*b*^ (mol%)	Select.^*b*^ (mol%)
1	None	7	99
2	Isopropylamine	30	98
3	Butylamine	35	98
4	*tert*-Butylamine	44	97
5	Trimethylamine	50	97
6	Triethylamine	58	96
7^*c*^	*tert*-Butylamine	28	97
8	Triethanolamine	14	99

^*a*^Reaction conditions: 0.3 mmol of thioanisole, 0.03 mmol of amine additive, 40 mg of Degussa P25 TiO_2_, 300 W Xe lamp, 5 mL of CH_3_OH, *λ* > 400 nm, 0.1 MPa of O_2_, 5 h.

^*b*^Determined by GC-FID using chlorobenzene as the internal standard, conversion of thioanisole, selectivity of methyl phenyl sulfoxide.

^*c*^0.015 mmol of amine.

To further prove the catalytic nature of the amine, we varied the amount of TEA used in the reaction to study its effect on the selective aerobic oxidation of thioanisole on TiO_2_ under visible-light irradiation in more detail. The results are summarized in Table 3. We discovered that decreasing the amount of TEA lead to a drop in conversion to some extent (entries 1 and 2, Table 3) in comparison with the starting result (entry 3, Table 3). Increasing the amount of TEA did not lead to an apparent increase in conversion (entries 4 and 5, Table 3). These combined results prove that TEA truly acts as a redox catalyst to aid the photoredox process. It would be more convincing if we detected the TEA free radical cation proposed in Scheme 1. However, only time-resolved electron spin resonance spectroscopy can perform this task due the fleeting nature of this free radical,^56^ which is unfortunately beyond our instrumental capacity but needs special attention in the future investigation in this direction.

**Table 3:** The effect of the amount of TEA on the photocatalytic oxidation of thioanisole on TiO_2_ under visible-light irradiation^*a*^

Entry	Amount of TEA (mmol)	*n*_substrate_/*n*_TEA_	Conv.^*b*^ (mol%)	Select.^*b*^ (mol%)
1	0.01	30	43	98
2	0.015	20	49	97
3	0.03	10	58	96
4	0.3	1	59	96
5	0.6	0.5	62	95

^*a*^Reaction conditions: 0.3 mmol of thioanisole, 0.1 MPa of O_2_, 40 mg of Degussa P25 TiO_2_, 300 W Xe lamp, 5 mL of CH_3_OH, 5 h, *λ* > 400 nm.

^*b*^Determined by GC-FID using chlorobenzene as the internal standard, conversion of thioanisole, selectivity of methyl phenyl sulfoxide.

**Fig. 2:**
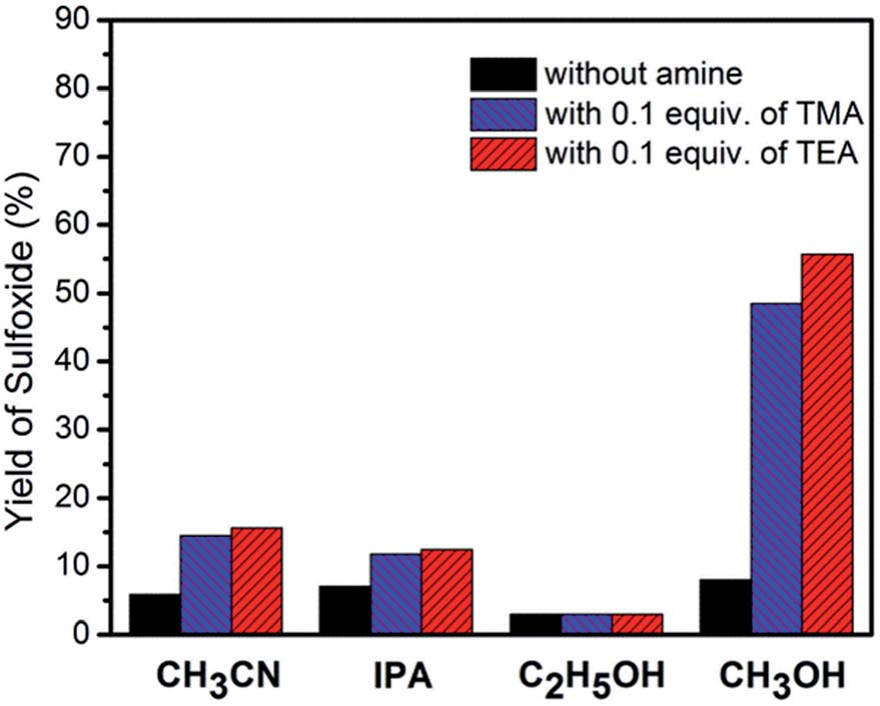
The influence of the solvent on the visible-light-induced selective oxidation of thioanisole with O_2_ on TiO_2_ in the presence of tertiary amine (TMA or TEA).

As previously stated, TMA or TEA are not stable molecules under typical photocatalytic aerobic oxidation conditions. However, they may be able to act as a redox mediator for the oxidation of thioanisole on TiO_2_ under visible-light irradiation due to protection by the reaction medium, CH_3_OH. Moreover, the protons from CH_3_OH would aid in the formation of sulfoxide and regeneration of the TiO_2_ surface. To confirm this hypothesis, CH_3_CN, one widely used solvent in photoredox catalysis, and other protic solvents were investigated in the formation of sulfoxide, which is summarized in Fig. 2. We can see that TMA and TEA can initiate the formation of sulfoxide in most of the tested solvents. However, the amine molecules were either partially (in the case of IPA and C_2_H_5_OH) or completely (in the case of CH_3_CN) consumed during the oxidation process. The protic nature of CH_3_OH is also stronger than that of IPA or C_2_H_5_OH, which aids in the formation of the product. The combination of these two factors enables CH_3_OH to confer the best result in Fig. 2.

We have established that the interaction between TEA and TiO_2_ induces the visible-light activity and further mediates the selective oxidation of sulfide into sulfoxide using O_2_. The choice of solvent, CH_3_OH, keeps TEA stable and ensures the selective and smooth formation of sulfoxide. Having obtained the optimal conditions for visible-light-induced photocatalytic aerobic oxidation, we proceeded to explore the scope of the sulfide substrates in Table 4. The stability of TEA in the photocatalytic system is reflected by the turnover number of 32 for the oxidation of thioanisole (entry 1, Table 4) with 78% of TEA preserved. To our delight, we found that thioanisole and substituted thioanisoles could be conveniently transformed into the corresponding sulfoxides with high selectivities (entries 2–9, Table 4). More than 90% of TEA could be recovered after the reaction in all cases. The substituted groups did not influence the results significantly in terms of reaction time and selectivity. However, it should be noted that the reaction times are slightly shorter and the selectivities are slightly higher for substituted thioanisoles with electron donating groups (entries 3–6, Table 4) than substituted thioanisoles with electron withdrawing groups (entries 7–10, Table 4). Very strong electron withdrawing groups, such as –NO_2_, make the reaction rate even slower (entry 10, Table 4). The –NO_2_ group could also undermine the stability of TEA in the reaction medium. However, increasing the amount of TEA could yield a higher conversion of sulfide (entry 11, Table 4). Meanwhile, replacing the methyl group with an ethyl group in thioanisole had a minor impact on the conversion of the sulfide (entry 12, Table 4). This is surprising as the photocatalytic aerobic oxidation of aliphatic substrates on TiO_2_ tends to yield undesirable selectivities due to the radical nature of the reaction process. In our reaction system, the solvent CH_3_OH could tame the uncontrollable aliphatic chain reaction, thus ensuring high selectivity. When the methyl group of thioanisole was replaced by a phenyl group, a significant decrease in conversion was observed (entry 13, Table 4), and a much longer reaction time was required to obtain a conversion comparable to that of thioanisole.

**Table 4:** Visible-light-induced oxidation of sulfides into sulfoxide with O_2_ on TiO_2_ with TEA as redox mediator^*a*^

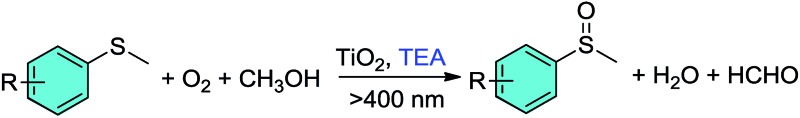					
Entry	Substrate	Product	*T* (h)	Conv.^*b*^ (mol%)	Select.^*b*^ (mol%)
1^*c*^	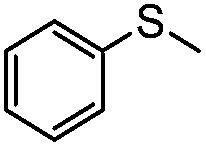	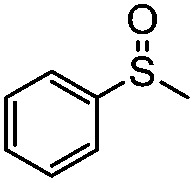	22	81	93
2	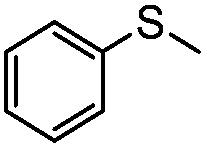	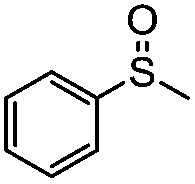	10	84	92
3	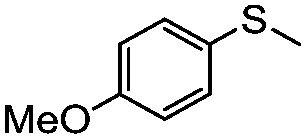	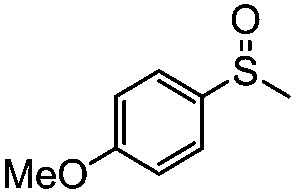	10	85	93
4	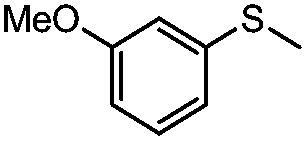	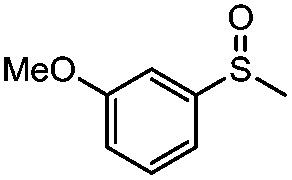	10	82	95
5	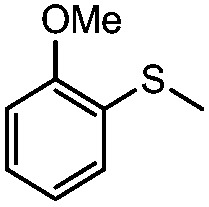	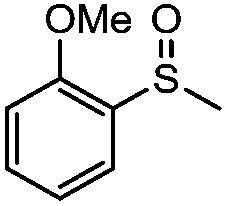	10	81	98
6	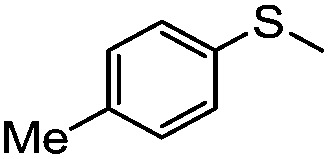	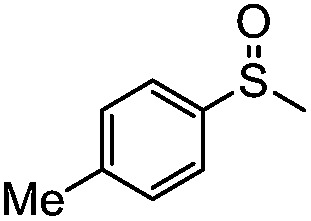	10	76	95
7	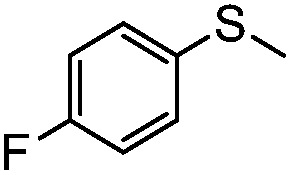	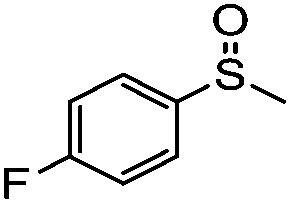	12	81	88
8	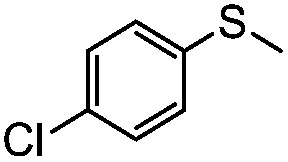	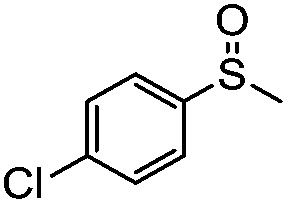	12	84	86
9	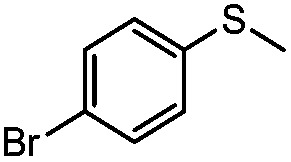	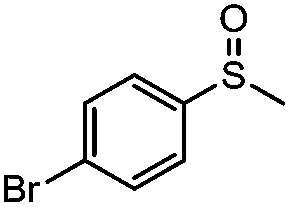	12	86	85
10	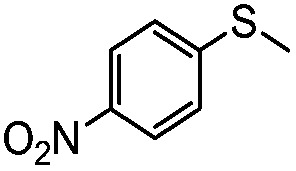	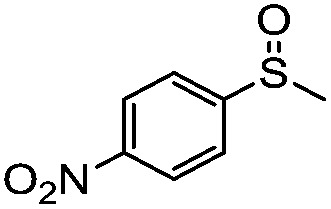	12	33	92
11^*d*^	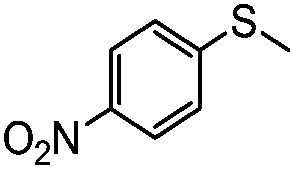	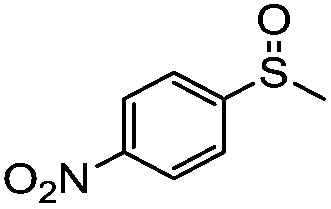	12	62	79
12	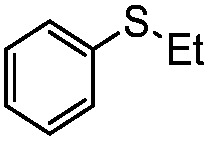	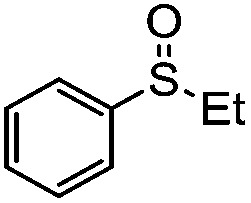	12	77	88
13	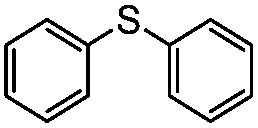	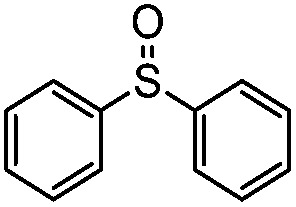	12	50	80

^*a*^Reaction conditions: 0.3 mmol of sulfide, 0.03 mmol of TEA, 40 mg of Degussa P25 TiO_2_, 300 W Xe lamp, 5 mL of CH_3_OH, *λ* > 400 nm, 0.1 MPa of O_2_.

^*b*^Determined by GC-FID using chlorobenzene as the internal standard, conversion of sulfide, selectivity of the corresponding sulfoxide.

^*c*^0.6 mmol of thioanisole, 0.015 mmol of TEA.

^*d*^0.1 mmol of TEA.

## Conclusions

In conclusion, through the photoredox synergistic catalysis of TiO_2_ and TEA, the visible-light-induced selective oxidation of sulfide into sulfoxide using O_2_ was successfully conducted in CH_3_OH. The interaction between TiO_2_ and TEA gives rise to the visible-light activity in the reaction system. Computational calculations have also revealed that TEA adsorbs more favourably onto the rutile surface. TEA acts as a redox mediator for the oxidation of sulfide, by donating electrons to the conduction band of rutile, which, *via* the conduction band of anatase, ultimately reach the final electron acceptor. Apart from its importance in synthesis, this finding could have implications on our understanding of the unique robust photocatalytic activity of Degussa P25 TiO_2_. However, we should admit that very limited visible-light was captured by the current system. A longer wavelength of light (*λ* > 420 nm and *λ* > 450 nm) should also be used for the reaction. We anticipate this could be achieved by anchoring a dye molecule onto TiO_2_ with a TEA-like redox mediator channelling the electron flow. Guided by the general strategy of photoredox synergistic catalysis, more versatile and complicated visible-light-induced organic transformations could be envisioned by adopting the surface of metal oxides as the reaction podium.
