# Supplementary material for: Tertiary amine mediated aerobic oxidation of sulfides into sulfoxides by visible-light photoredox catalysis on TiO2
Source: Chem Sci. 2015 Jun 9;6(8):5000–5. doi: 10.1039/c5sc01813g (PMC5664354; doi:10.1039/c5sc01813g)
Supplement: Supplementary file 1 [file SC-006-C5SC01813G-s001.pdf]

Electronic Supplementary Material (ESI) for Chemical Science.
This journal is © The Royal Society of Chemistry 2015

Electronic Supplementary Information

Table of contents

Reagents and solvents: S2

General procedure for the oxidation of sulfide: S2

Instrumentation and conditions: S2

Light source: S2

UV-Vis: S3

XPS: S3

GC and GC-MS: S3

DFT calculation: S4

Figure S1: XPS spectroscopy of Degussa P25 TiO_2 without adsorption of TEA (black line) and after adsorption of TEA (red line) S3

Figure S2: The side view and top view of the fixed-double-layer (FDL) model of (2×1) rutile (110) and (2×2) anatase (101) surfaces. The bottom two layers (inside the dotted rectangle zone) were fixed to their bulk positions during geometric optimizations. S5

Figure S3: The most stable structures with TMA on the surface. The numbers are the distances from N atoms to its nearest Ti atoms S5

Figure S4: DOS of TMA on rutile TiO_2 (110) by PBE S6

Figure S5: GC-FID results for Table 4 .... S7-S13

Reagents and solvents:

All of the reagents used were obtained from commercial suppliers such as Sigma-Aldrich, Alfa Aesar and TCI, etc. The solvents were supplied by Merck or Fischer Scientific. All the regents and solvents are of the highest purity and used without further purification.

General procedure for the oxidation of sulfide:

In a typical reaction, 40 mg of Degussa P25 TiO_2, 0.3 mmol of thioanisole and 0.01 mmol of triethyamine were added to 5 mL of CH_3OH in a Pyrex vessel. After the reaction mixture was stirred for 30 min in dark to reach adsorption equilibrium, O_2 was purged into the Pyrex vessel to raise the initial pressure to 0.1 MPa. The reaction mixture was magnetically stirred at 800 r/min and illuminated with λ>400 nm visible light irradiation in an air-conditioned room to warrant the reaction temperature constantly at 25 $^{\circ}$C. At the end of reaction, the TiO_2 photocatalyst particles were separated from the reaction mixture by filtration and the products were quantitatively analyzed by gas chromatography (GC) equipped with a flame ionization detector (FID) using chlorobenzene as the internal standard. The structure of products were confirmed by comparison with the retention time with standard samples and further confirmed by gas chromatography–mass spectrometry (GC-MS).

Instrumentation and conditions:

Light source:

The reaction was irradiation with an Asahi Spectra MAX-303 300 W Xenon light source using a UV-VIS mirror model. In this mirror model, the irradiating wavelength range is 270 nm-650 nm, thus the possible heating of the reaction medium the infrared light is completely excluded. Additional Asahi Spectra longpass cutoff filters (>400 nm) are used to control irradiation wavelength range during the reaction. The reaction medium was maintained at room temperature throughout the experimental process.

UV-Vis:

The UV-visible absorption spectra of the solid samples were recorded on a Shimadzu UV 2550 UV-visible Spectrophotometer with a diffuse reflectance measurement accessory.

XPS:

X-ray Photoelectron Spectroscopy (XPS) were measured by an ESCALAB250XI. The incident radiation was Mg Kα X-ray (1253.6 eV) at 400 W and a charge neutralizer was turned on for acquisition. The binding energy of N1s was corrected by C 1s peak (284.8 eV) from residual carbon.

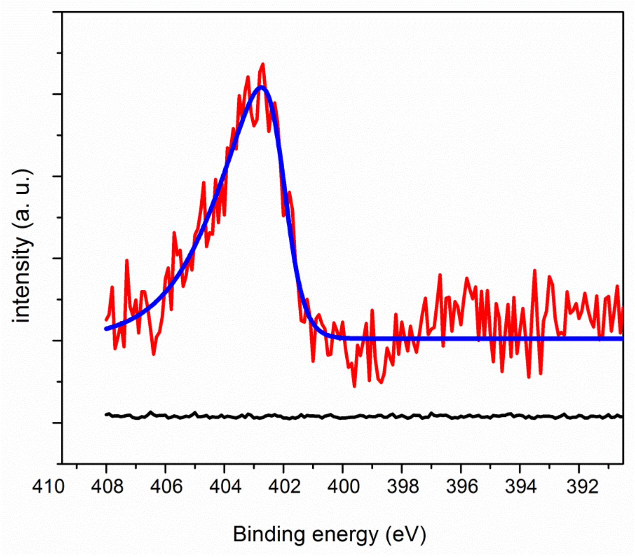

Figure S1: XPS spectroscopy of Degussa P25 TiO_2 without adsorption of TEA (black line) and after adsorption of TEA (red line)

GC and GC-MS:

The quantitative measurements of conversions of substrate and selectivities of products were made on an gas chromatography (Agilent 7890A) equipped with a flame ionization detector (FID) and Agilent Technology 19091J-413 capillary column (30 m×0.32 mm×0.25 μm) using high pure N_2 as the carrier gas. Standard analysis conditions: injector temperature 250 ˚C, detector temperature 300 ˚C, column temperature program: 50 ˚C (hold 1.5 min) raised up to 300 ˚C (hold 3 min) at a rate of 20 ˚C/min. Gas chromatography–mass spectrometry analysis was performed on a Shimadzu GC 2010 gas

chromatograph equipped with a Shimadzu GCMS-QP2010 Ultra electron ionization mass spectrometer using a Restek (Rxi®-5Sil MS) capillary column (30 m×0.25 mm×0.25 μm) with high pure He as the carrier gas.

DFT calculation:

The first-principle calculations were performed using the Vienna ab initio simulation package (VASP)[1] that based on the density functional theory. The projector augmented wave (PAW)[2] method was used to describe the electron–ion interaction and the exchange correlation between electrons was described by the generalized gradient approximation (GGA) in the Perdew–Burke–Ernzerhof (PBE) form. We used a cutoff energy of 500 eV for the plane-wave basis set. Spin polarization was allowed for all systems. The volume of the supercell was fixed but all the internal freedoms were fully relaxed. For the bulk rutile and anatase TiO_2, the (4×4×6) and (6×6×2) *k*-mesh within the Monkhorst–Pack scheme were used respectively. Lattice relaxation was continued until the forces on all the atoms were converged to less than 10^{-2} eV $Å^{-1}$. The lattice constants reproduced from GGA-PBE computations, as well as the theoretical and experimental values from the literatures are listed in Table S1. Our results are in good agreement with both the previous DFT results and experimental results.

Based on our calculated lattice parameters (Table S1), slab models for rutile (110) and anatase (101) surfaces were derived using the fixed-double-layer (FDL) approach[3] with the two bottom layers of the slab fixed to their bulk positions (Figure S2, see supporting information for more details). To accommodate the TMA molecule on TiO_2 surface, a (2×1) supercell rutile (110) slab, which contains 4 TiO_2-layers and 48 atoms, and a (2×2) supercell anatase (101) slab, which contains 4 TiO_2-layers and 96 atoms, were adopted. The (4×4×1) k-mesh generated by the Monkhorst-Pack method was used to optimize the geometry for both slabs. The calculated rutile (110) surface formation energy is 0.51 J/m^2, which is in excellent agreement with reported result (0.52 J/m^2)[4] and the calculated anatase (101) surface formation energy is 0.52 J/m^2, which is also consistent with the reported result (0.49 J/m^2).[5]

Table S1 The calculated lattice parameters (Å) for rutile and anatase TiO_2, as well as the TMA. Other theoretical and experimental values are also listed.

	rutile		anatase		TMA	
	a	c	a	c	d_{N-C}	d_{C-H}
This work	4.660	2.969	3.821	9.697	1.454	1.099
Other DFT	4.670[4]	2.971[4]	3.786[5]	9.737[5]	1.457[6]	1.095[6]
Expt.	4.587[6]	2.954[6]	3.782[6]	9.502[6]	1.458	

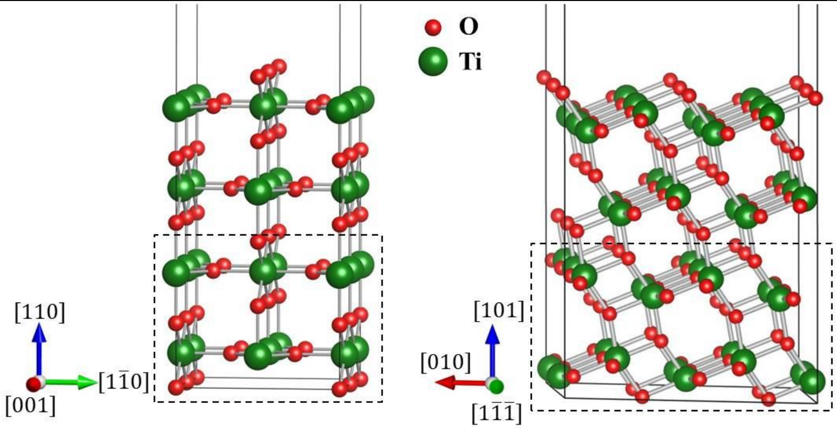

Figure S2: The side view and top view of the fixed-double-layer (FDL) model of (2×1) rutile (110) and (2×2) anatase (101) surfaces. The bottom two layers (inside the dotted rectangle zone) were fixed to their bulk positions during geometric optimizations.

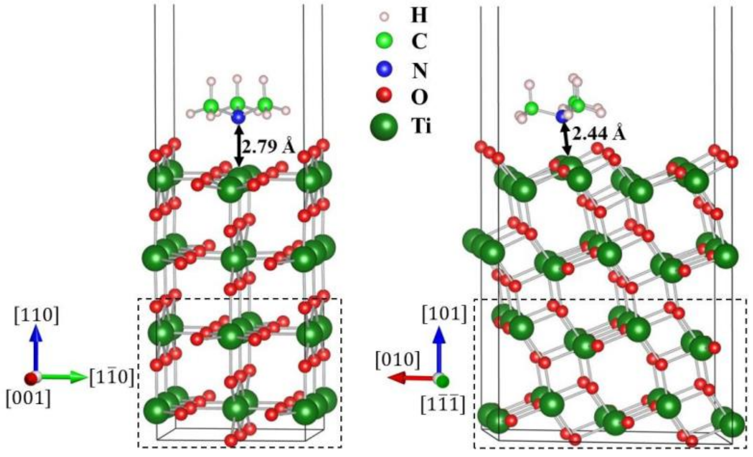

Figure S3: The most stable structures with TMA on the surface. The numbers are the distances from N atoms to its nearest Ti atoms.

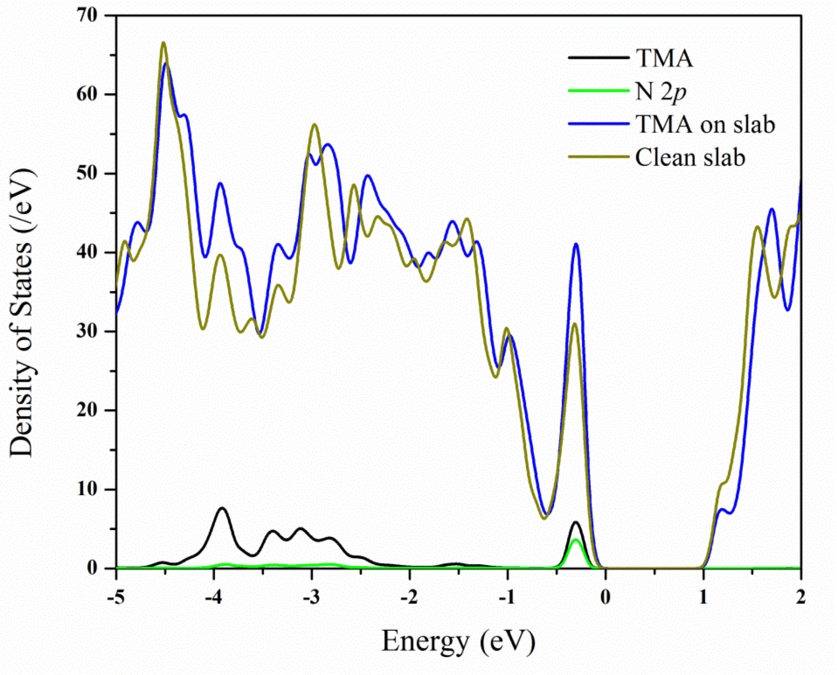

Figure S4: DOS of TMA on rutile TiO2 (110) by PBE.

[1] G. Kresse, J. Furthmüller, *Phys. Rev. B* **1996**, *54*, 11169-11186.

[2] J. P. Perdew, K. Burke, M. Ernzerhof, *Phys. Rev. Lett.* **1996**, *77*, 3865-3868.

[3] K. J. Hameeuw, G. Cantele, D. Ninno, F. Trani, G. Iadonisi, *J. Chem. Phys.* **2006**, *124*, 024708.

[4] R. Tonner, *ChemPhysChem* **2010**, *11*, 1053-1061.

[5] M. Lazzeri, A. Vittadini, A. Selloni, *Phys. Rev. B* **2001**, *63*, 155409.

[6] J. K. Burdett, T. Hughbanks, G. J. Miller, J. W. Richardson, J. V. Smith, *J. Am. Chem. Soc.* **1987**, *109*, 3639-3646.

Figure S5: GC-FID results for Table 3. The results were all obtained using a split mode: for entry 1, the split ratio is 1/30; for entries 2-13, the split ratio is 1/20.

Table 4, entry 1

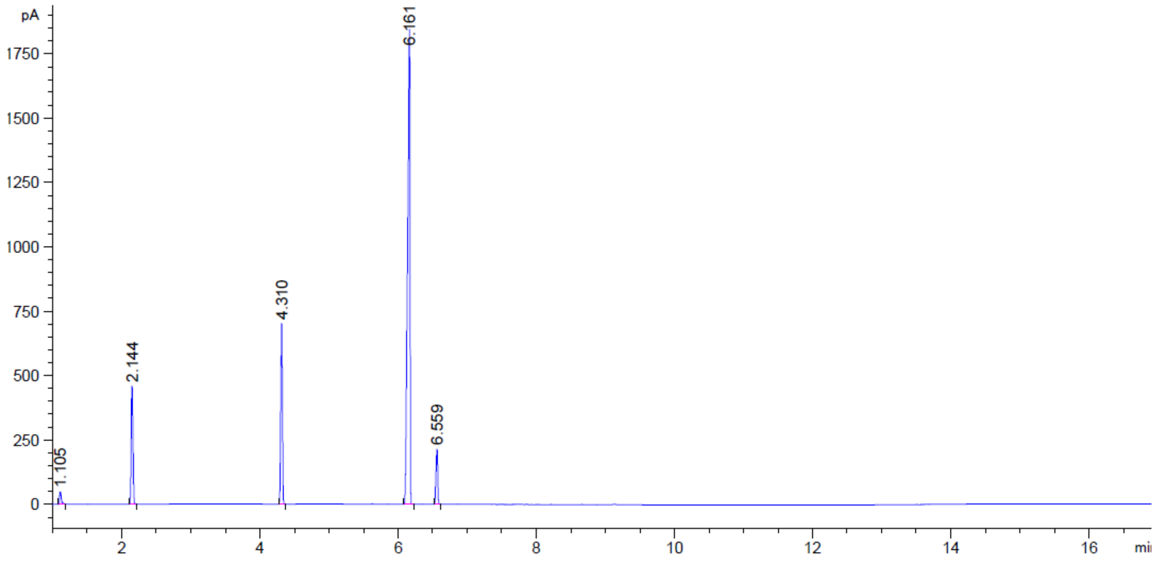

Retention time (min)	1.105	2.144	4.310	6.161	6.559
Chemical	$N(C_2H_5)_3$	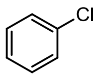	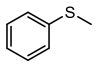	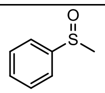	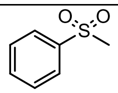

Table 4, entry 2

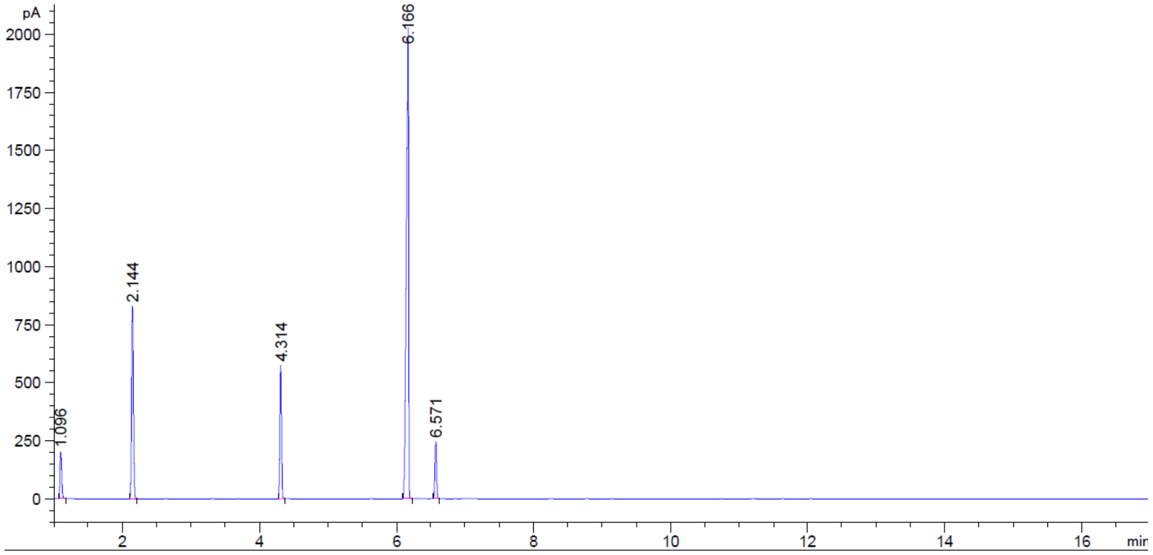

Retention time (min)	1.096	2.144	4.314	6.166	6.571
Chemical	$N(C_2H_5)_3$	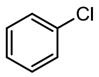	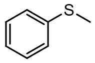	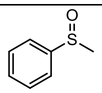	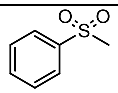

Table 4, entry 3

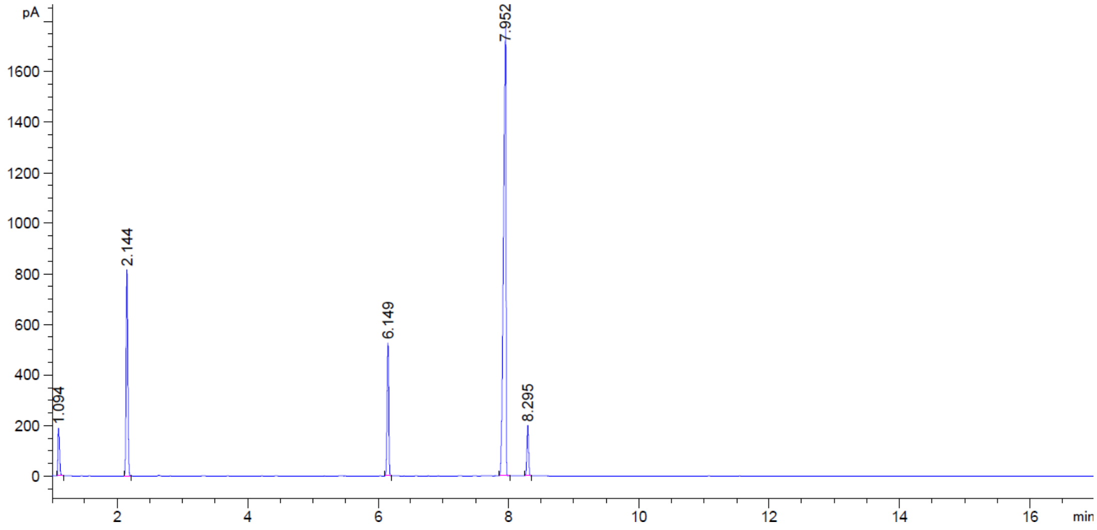

Retention time (min)	1.094	2.144	6.149	7.952	8.295
Chemical	$N(C_2H_5)_3$	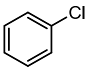	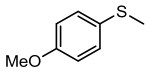	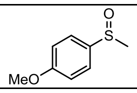	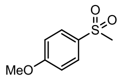

Table 4, entry 4

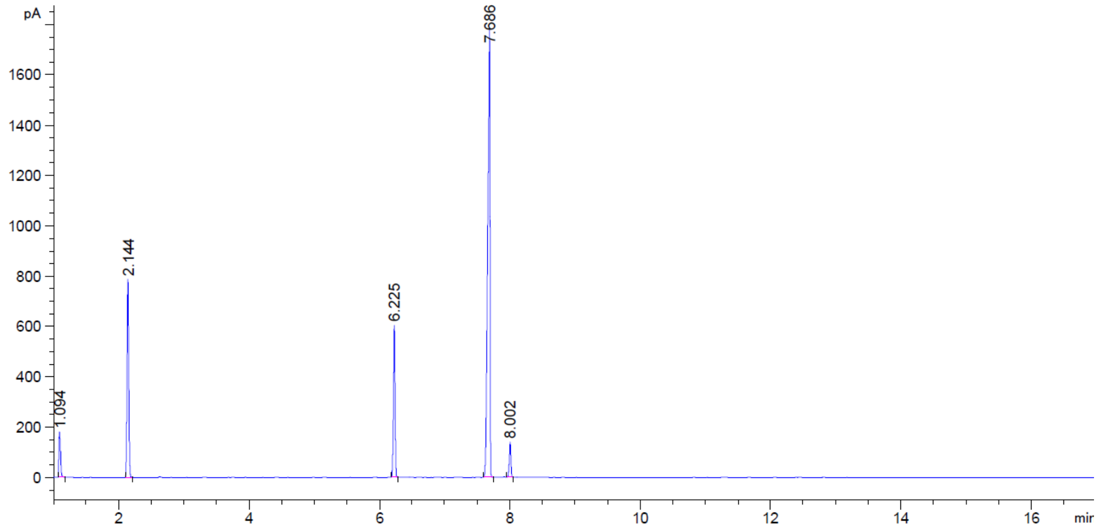

Retention time (min)	1.094	2.144	6.225	7.686	8.002
Chemical	$N(C_2H_5)_3$	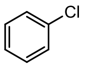	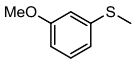	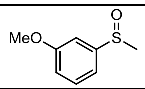	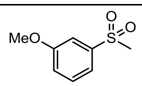

Table 4, entry 5

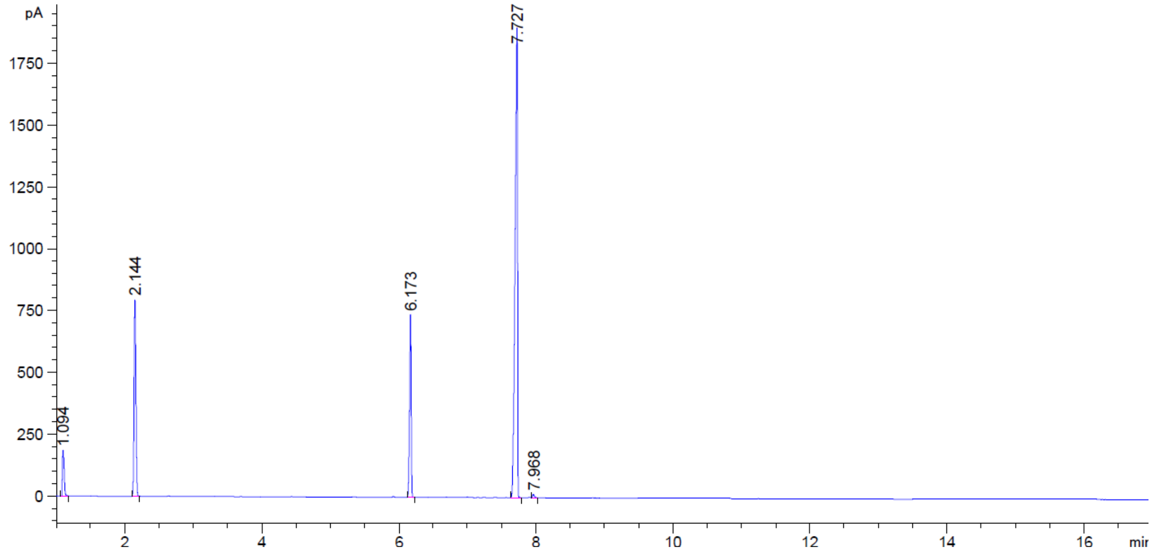

Retention time (min)	1.094	2.144	6.173	7.727	7.968
Chemical	$N(C_2H_5)_3$	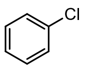	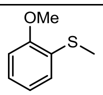	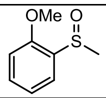	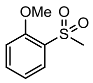

Table 4, entry 6

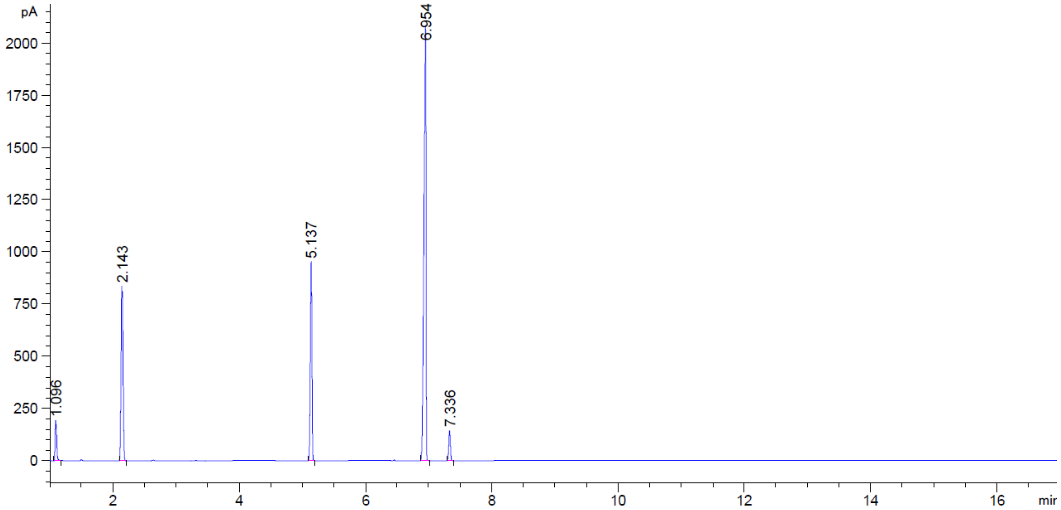

Retention time (min)	1.096	2.143	5.137	6.954	7.336
Chemical	$N(C_2H_5)_3$	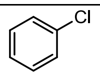	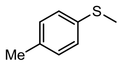	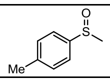	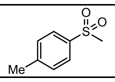

Table 4, entry 7

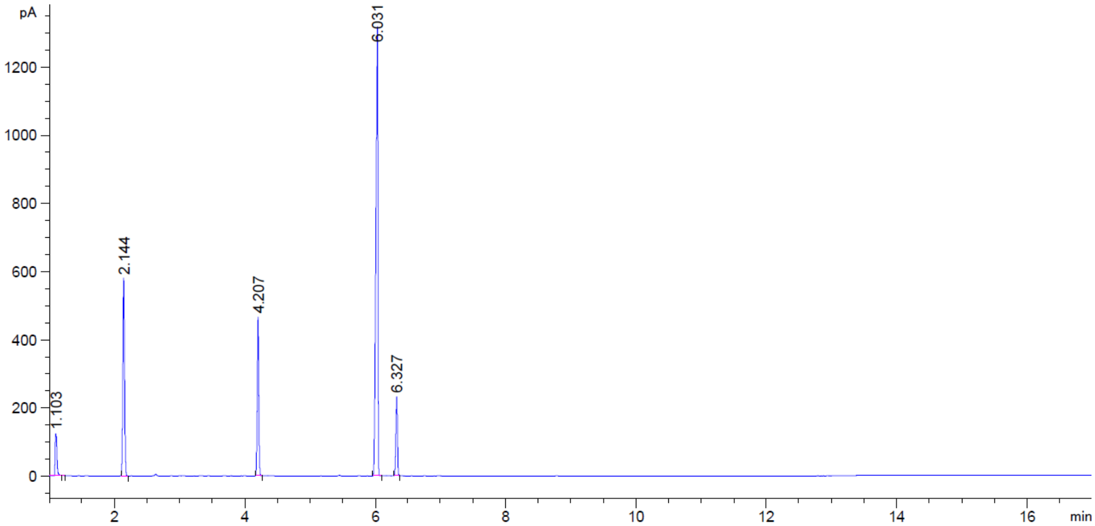

Retention time (min)	1.103	2.144	4.207	6.031	6.327
Chemical	$N(C_2H_5)_3$	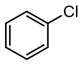	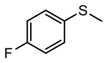	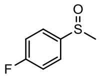	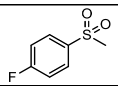

Table 4, entry 8

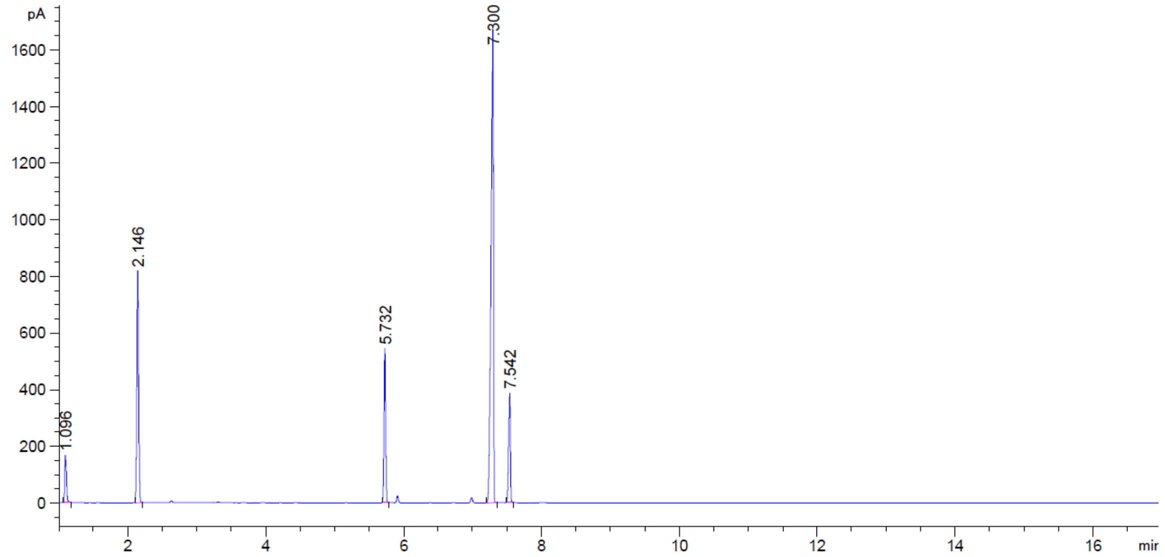

Retention time (min)	1.096	2.146	5.732	7.300	7.542
Chemical	$N(C_2H_5)_3$	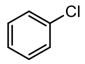	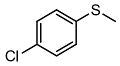	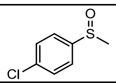	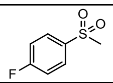

Table 4, entry 9

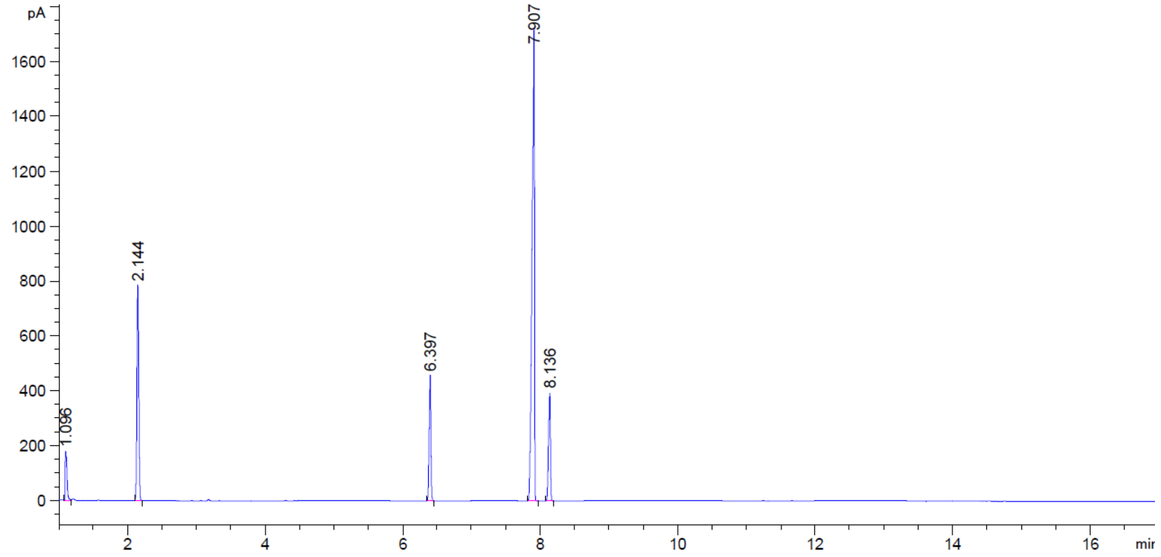

Retention time (min)	1.096	2.144	6.397	7.907	8.136
Chemical	$N(C_2H_5)_3$	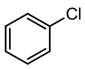	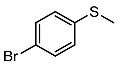	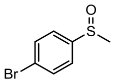	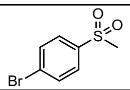

Table 4, entry 10

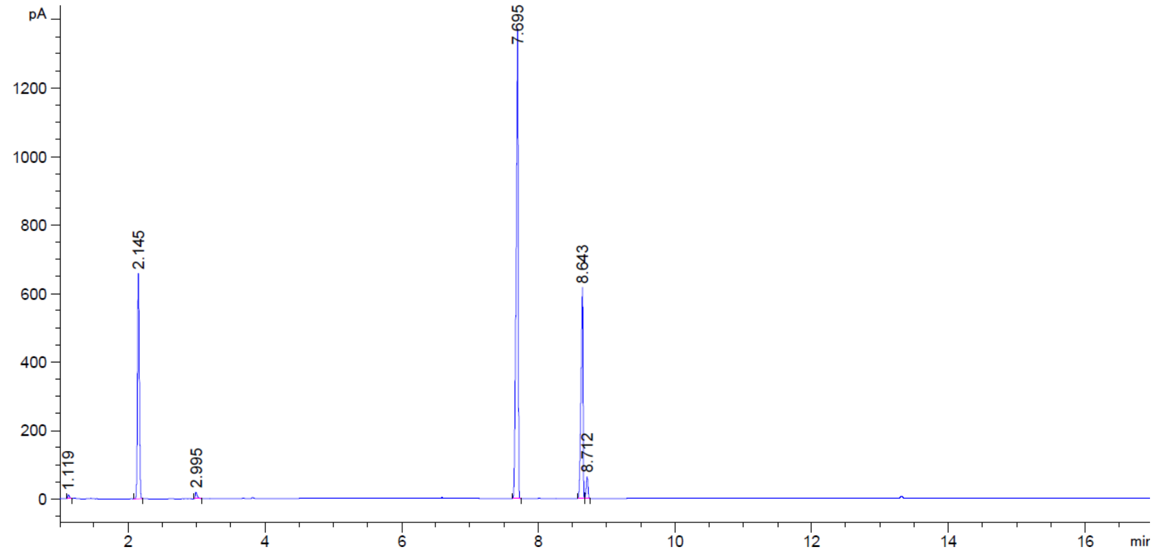

Retention time (min)	1.119	2.145	7.695	8.643	8.712
Chemical	$N(C_2H_5)_3$	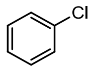	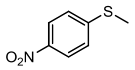	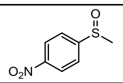	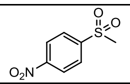

Table 4, entry 11

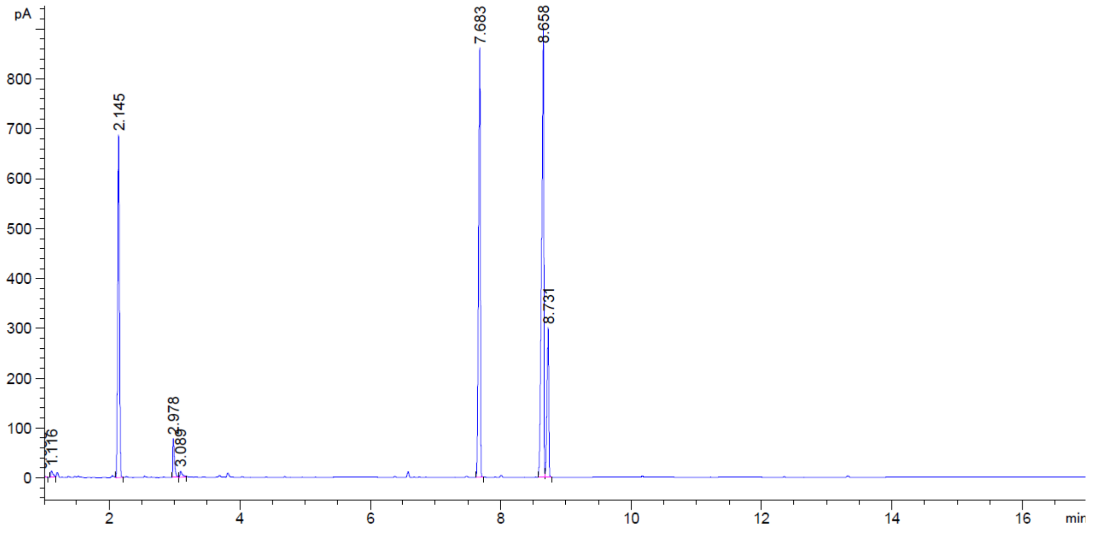

Retention time (min)	1.116	2.145	7.683	8.658	8.731
Chemical	$N(C_2H_5)_3$	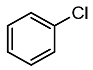	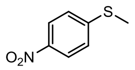	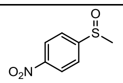	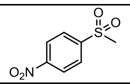

Table 4, entry 12

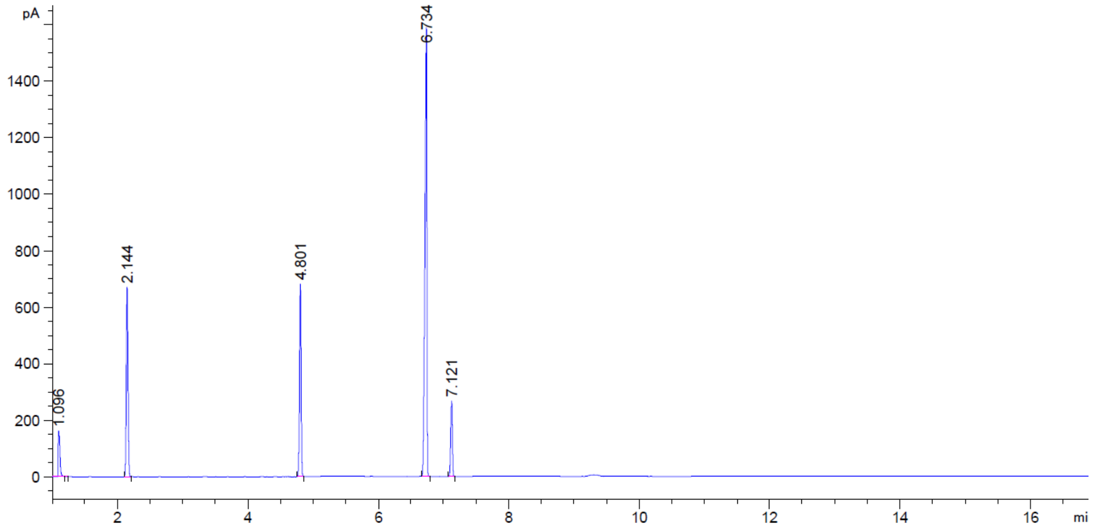

Retention time (min)	1.096	2.144	4.801	6.734	7.121
Chemical	$N(C_2H_5)_3$	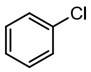	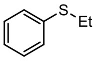	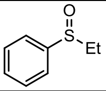	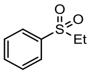

Table 4, entry 13

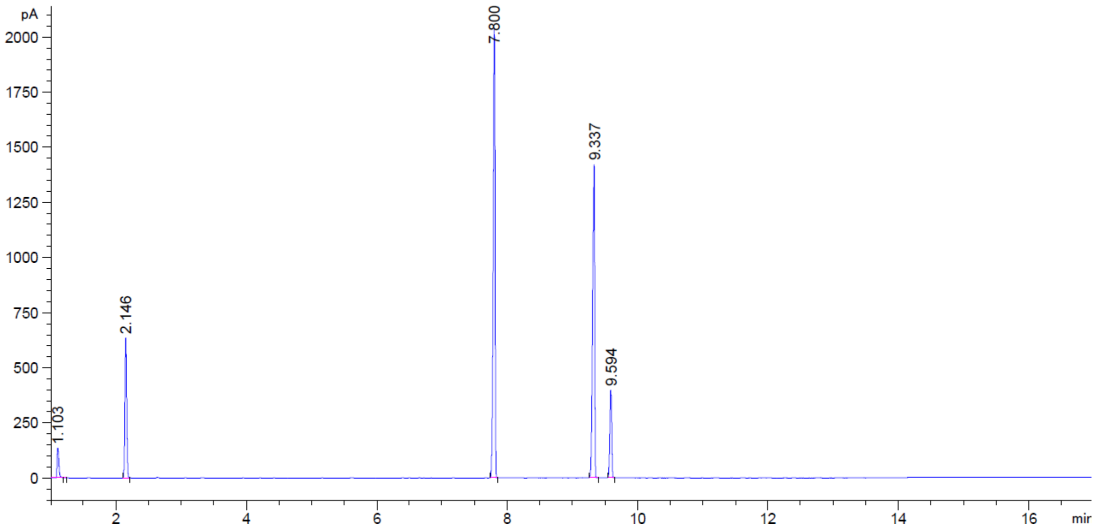

Retention time (min)	1.103	2.146	7.800	9.337	9.594
Chemical	$N(C_2H_5)_3$	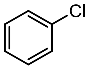	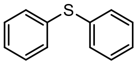	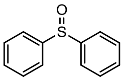	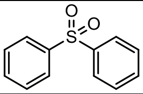
